# Genome Sequence of *Enterococcus mundtii* EM01, Isolated from *Bombyx mori* Midgut and Responsible for Flacherie Disease in Silkworms Reared on an Artificial Diet

**DOI:** 10.1128/genomeA.01495-17

**Published:** 2018-01-18

**Authors:** Beatriz de Diego-Diaz, Laura Treu, Stefano Campanaro, Vinicius da Silva Duarte, Alessio Saviane, Silvia Cappellozza, Andrea Squartini

**Affiliations:** a Department of Environmental Engineering, Technical University of Denmark, Kongens Lyngby, Denmark; b Department of Chemistry, University of Navarra, Navarra, Spain; c Department of Biology, University of Padua, Padua, Italy; d Department of Microbiology, Federal University of Viçosa, Viçosa, Brazil; e CREA Centro di Ricerca Agricoltura e Ambiente, Padua, Italy; f Department of Agronomy, Food, Natural Resources, Animals and Environment (DAFNAE), Legnaro, PD, Italy

## Abstract

The whole genome sequence of *Enterococcus mundtii* strain EM01 is reported here. The isolate proved to be the cause of flacherie in *Bombyx mori*. To date, the genomes of 11 other *E. mundtii* strains have been sequenced. EM01 is the only strain that displayed active pathological effects on its associated animal species.

## GENOME ANNOUNCEMENT

*Enterococcus mundtii* is a taxon stemming from the reclassification of different enterococcal bacteria that formerly included *Streptococcus* (*Enterococcus*)* faecalis* and *Enterococcus faecium* (1). Its role as a potential pathogen has been signaled (2–4), as has its apparently commensal presence within the guts of different vertebrate and invertebrate animals (5). Being capable of lactic fermentation, its occurrence in fermented dairy products is also documented (6, 7).

There are already 11 available genome sequences from this species. The sequenced strains were isolated from different sources and are QU25 (8, 9), EMB156 (GenBank accession number NZ_CP022340), ATCC 882 (10), CRL1656 (11), CRL35 (6), QAUEM2808 (7), SL_16 (12), DSM 4838 (NZ_JXKV01000001), CGB1038-1_S1 (NZ_MSTR01000001), 6B1_DIV0119 (NZ_NGMS01000001), and C2 (NZ_FOUC01000001). The genome sequences are complete for two of these strains, QU25 and EMB156.

Strain EM01 was isolated in Italy in 2008 from 5th-instar larvae of the silkworm, *Bombyx mori*, reared on a nonsterile artificial diet based on mulberry leaves (13). EM01 was shown to be the causal agent of flacherie disease in silkworms. The only other available genome sequence of an *E. mundtii* isolate from the same host (strain EMB156) came instead from the guts of healthy *B. mori* silkworms reared in China. In fact, reports of *E. mundtii* isolation from silkworms in that country judged its occurrence as potentially beneficial for the same host (14). This makes the present report particularly relevant, as it allows inspection of whether distinctive genetic traits could possibly differentiate an ascertained pathogenic version of the taxon from its supposedly nonthreatening counterpart.

An Illumina MiSeq sequencer (Ramaciotti Centre, Australia) was used for the whole-genome sequencing of strain EM01. The genomic libraries used for this purpose were obtained with the Nextera XT kit (Illumina, Inc., USA). Up to 1,918,184 paired-end reads (2 × 250 bp) were generated, and these yielded 153-fold coverage of the studied genome. The assembly of 99.4% of these reads generated 54 scaffolds. All the aforementioned analyses were performed with version 10.1.1 of the CLC Genomics Workbench software (Qiagen Bioinformatics, Germany) (5). The draft genome of *E. mundtii* EM01 is 3,134,567 bp in length, with a mean G+C content of 38.2%. The *N*_50_ for this isolate reached 136,907 bp.

Rapid Annotations using Subsystems Technology (RAST) (15) was used for genome annotation, and 2,994 coding sequences were detected, as were 53 structural RNAs. Up to 51 of the genes detected are related to virulence, disease, and defense, and 33 are phage-associated sequences. No clusters of regularly interspaced short palindromic repeats were detected. When comparing the sequence of this genome with the other whole-genome sequences reported in the literature, it can be seen how the gene identification for EM01 is slightly better than that for all others except QU25 (3,229).

Finally, a BLAST search was performed using the 16S rRNA gene sequence, and 100% identity and query coverages were found with *E. mundtii* ATCC 43186 (GenBank accession number NR_024906). The genome sequence was used as input for PHASTER software. PHASTER analysis yielded two intact and four incomplete prophage regions, which is consistent with analysis results from the other reference genomes.

### Accession number(s).

This whole-genome shotgun project has been deposited at DDBJ/ENA/GenBank under the accession number PHIL00000000. The version described in this paper is version PHIL01000000.
